# The construct of sexual openness for females in steady intimate relationships

**DOI:** 10.1371/journal.pone.0172274

**Published:** 2017-06-21

**Authors:** Diana Rausch, Arne Dekker, Martin Rettenberger

**Affiliations:** 1 Department of Psychology, Johannes Gutenberg-University Mainz (JGU), Mainz, Germany; 2 Institute for Sex Research and Forensic Psychiatry, University Medical Center Hamburg-Eppendorf, Hamburg, Germany; 3 Centre for Criminology, Wiesbaden, Germany; Indiana University School of Medicine, UNITED STATES

## Abstract

The analysis of open-minded attitudes towards sexuality in general requires a construct based on attitudinal dimensions. Although several existing studies involve sexual attitudes, they differ substantially and standardized conceptual work is missing. Thus, the authors introduce the latent variable sexual openness to develop a construct based on self-oriented attitudes towards different sexual topics. Available survey data of female German students in a steady relationship allowed providing a first empirical test for the applicability of this construct. Five subdimensions are acknowledged central for sexual openness: sexual practices, masturbation, bisexuality, permissiveness, and pornography consumption. Confirmatory factor analysis and correlations confirmed the idea of an underlying mechanism with an impact on all five variables. Though further validation of the construct of sexual openness is required, the findings strongly support the notion of an overarching latent attitude variable, which influences the individual relation to everything sexual. The results were compared to other studies and potential approaches for future analyses were proposed.

## Introduction

Sexual well-being is known to have positive effects on mental health, self-confidence, quality of partnership, and body image [1–6]. Similarly, sexual dysfunction is associated with a reduced quality of life. This is due to the impact on depression, anxiety, and distressed feelings [7–9]. An increased level of sexual well-being is connected to open attitudes towards sexuality [10–12]. These attitudes are influenced by the complexity of personal experience, as well as the cultural and social environment [13–22]. Because of its apparent impact on the quality of life we introduce the construct sexual openness for systematic research and model development of sexual functioning.

### Previous research

Although no previous research used sexual openness as proposed in this study there are several works, which tap parts of the construct. Many different measures of individual dispositions towards sexuality have been proposed [23–25]. In the following erotophilia-erotophobia and the concept of sexual liberalism serve as the main theoretical links of sexual openness to previous research in this field.

Erotophilia-erotophobia is defined as “the disposition to respond to sexual cues along a negative-positive dimension of affect and evaluation” [26, 27]. Since this concept was operationalized with the Sexual Opinion Survey (SOS) it has been used as a basis for research on several sexual issues. This includes topics such as sexual arousal, reactions to sexual stimuli, body image, and masturbation [9, 28–33].

An advantage of the SOS is the measurement of a diversity of sexual topics. Although erotophilia-erotophobia consists of affective responses to sexual cues, it is often used to characterize sexual attitudes. The correlation of the SOS scores and sexual attitudes or behavior are just weak to moderate [34], which emphasizes the requirement of new integrated constructs and corresponding measurement tools.

Updates of the SOS partially address the mentioned aspects but mostly from a measurement perspective. They tend to concentrate on single subdimensions (e.g., Sexual Anxiety Scale, [35]; emphasize on pornography and practices) or societal norms related to sexuality, which strongly depend on the cultural environment (e.g. Sexual Attitude Scale, [36]: *Premarital sex may be a sign of a decaying social order*, *I think sex should be reserved for marriage*). Some scales present a confused classification of items (e.g., Sexual Attitudes Scale, [37]: sexual practices measured as a mixture of birth control, sex education, sex toy usage and masturbation) or focus on a few aspects of sexual liberalism (e.g., Inventory of Dyadic Heterosexual Preferences, [38]: erotophilia measured by only eight items on specific practices and preferences; Sexual Attitudes Scale, see [37]: subdimension communion contains items measuring the satisfaction with and quality of sexuality).

However, most of these scales also have conceptual benefits. For example, the Sexual Attitudes Scale introduced by Hendrick and Hendrick [37] contains a comprehensive operationalization of the construct permissiveness, which is still largely up to date. Fallis et al. [35] criticize the SOS items and propose the Sexual Anxiety Scale as an alternative. They explained most of the variance in their data with a factor that mainly includes items about pornography, masturbation, sexual practices, and statements about reactions to possible embarrassing situations (e.g., overhearing other people having sex, seeing two people kissing or fondling each other). The Trueblood Sexual Attitudes Questionnaire consists of a compilation of items on attitudes towards others, and the same amount of items on individual attitudes, which are applicable today [39, 40].

In summary, the revision and adjustments of erotophilia-erotophobia and sexual liberalism mainly focus on the measurement while no major theoretical revisions of the original constructs have been conducted. This study aims to focus on the overarching construct behind attitudes towards sexuality, independent of the specific instruments used to measure it.

### The present study

**Fig 1:**
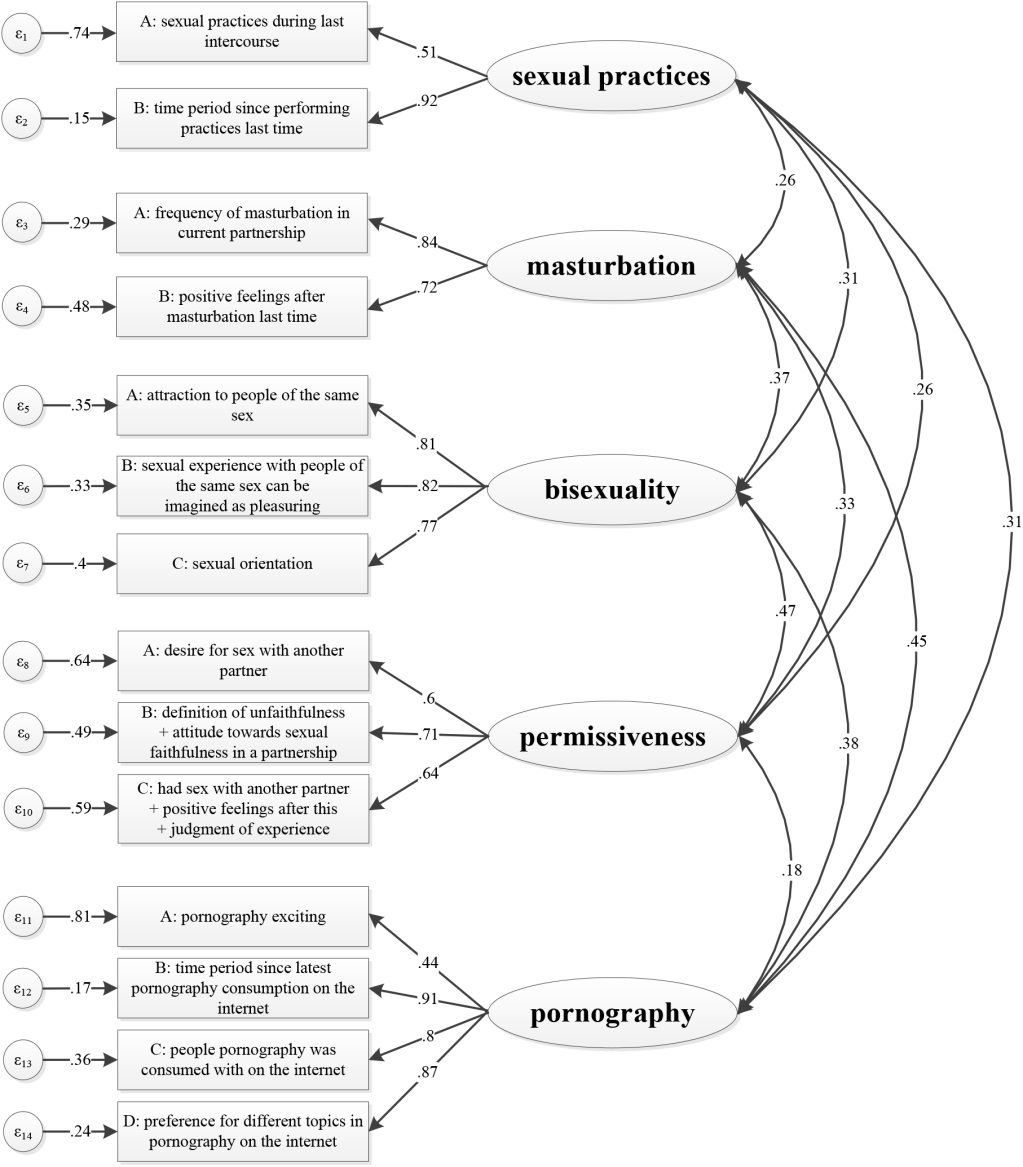
Confirmatory factor analysis for sexual openness. p < .001 for all estimated factor loadings.

The purpose of this study is to structure, rearrange, and add to the ideas of erotophilia and sexual liberalism to propose an attitude-based construct named *sexual openness*. The big five personality trait *openness to experience* is an important basis for the development of attitudes towards different sexual content and thus for the individual formation of sexual openness. However, sexual openness is more responsive to external influences and might change significantly over time. Previous research on openness towards sexual topics serves as a basis for the new integrated construct sexual openness. Individual attitudes towards masturbation, pornography, permissiveness, bisexuality, and sexual practices form the five subdimensions of this new latent variable. Positive attitudes towards masturbation indicate a higher level of acceptance for the own sexual desires [41, 42]. A liberal view of bisexuality and sexual practices signifies high interest in new sexual experiences [43]. We will also include permissiveness in our conceptualization of sexual openness [44]. Finally, sexuality-related media are able to provoke positive emotions or resentment [45–51]. Based on these relationships and their theoretical background, the five mentioned subdimensions should highly correlate and cover central aspects of individual sexual openness (see Fig 1). We will estimate their distinctiveness and investigate whether these aspects are part of one latent construct.

The statistical analysis employs data of a survey about sexuality in students conducted by the Institute for Sex Research and Forensic Psychiatry at the University Medical Center Hamburg-Eppendorf [52]. Due to its different focus, many of the items measured sexual behavior, which represents only the manifest part of sexual openness as an association of attitudes towards sexual topics. Thus we had to operationalize sexual openness relying on behavioral measures. However, the emphasis is on the underlying latent attitude independent of partnership and society.

Unfortunately, using items referring to sexual behavior has implications for the composition of the sample. It is expected that the sexuality of people in steady partnerships differs substantially from those without a partner [53–56]. For example, the lack of a sexual partner affects the frequency of sexual behavior. Thus, we had to exclude singles in our final sample, although their attitudes are supposed to align with the same construct. Additionally, attitudes towards several sexuality-related topics such as pornography or masturbation are unequal in men and women [5, 32, 57–59]. These findings imply gender differences in a global concept for sexual attitudes. Several studies estimated higher levels of sexual dissatisfaction for women than for men due to arousal and interest problems [2, 7, 60]. Thus, research on female sexuality is of particular importance and we decided to focus on women in this study. Of course the sexual openness of men and the expected differences to women should be covered in future analyses.

Therefore, the main goal of the statistical analysis is to provide evidence for the construct of sexual openness independent of the used measurement model. We expected interrelations of all subdimensions due to their connection to the latent construct sexual openness. We used confirmatory factor analysis (CFA) as statistical method and tested different estimation algorithms to account for the effect of non-normality and the sample size. As asymptotically distribution free (ADF) estimation is recommended for usage in case of non-normal distribution, a more accurate estimation was expected for ADF. This opposes the widespread tendency to use maximum likelihood estimation in many analyses [31, 61, 62].

## Method

### Participants

The used data derived from a survey, which was the fourth in a series of similar surveys since 1966 and part of a research project of the Institute for Sex Research and Forensic Psychiatry at the University Medical Center Hamburg-Eppendorf [52]. The aim of the project is to analyze the current state and changes in sexual behavior and attitudes of German students (for other publications related to this longitudinal study see, for example, [63–65]. The project was funded by the German Research Foundation (Deutsche Forschungsgemeinschaft, DFG). The study design was approved by the Data Protection Supervisor of the Free and Hanseatic City of Hamburg (federal state of Germany; approval number: D4/17.06-00/1). A further ethical statement according to the APA standards was included in the cover letter of the present manuscript. Written informed consent was not given by participants but the participants’ information was anonymized and de-identified prior to analysis. There was no clinical data or material used, i.e., there was no analysis of patients’ information. The data was collected at 15 universities located in different cities all-over Germany between May and December 2012. Participants answered with a response rate of nearly 28%, which resulted in a final sample of 2,081 German students consisting of 1,280 women and 796 men. The participants’ age ranged from 16 to 54 (*M* = 24.24, *SD* = 4.00). Of all respondents 63.74% (*n* = 1,327) indicated being in a relationship with a partner; however more female (66.87%) than male (58.79%) students affirmed this question. For our analyses, the group of interest consisted of women committed in a relationship, resulting in a final sample of *n* = 859. This data enabled us to conduct statistical analyses dependent on larger sample sizes, but we had to choose from predetermined items to construct our measurement model.

### Subdimensions

Under consideration of erotophilia and other concepts like sexual liberalism we constructed the model of sexual openness consisting of the five subdimensions sexual practices, masturbation, bisexuality, permissiveness, and pornography [27, 34, 35, 38, 39, 41, 66–69]. In order to measure these subdimensions, questions from the questionnaire sections about sexual experiences, sexuality in the new media, and sexual well-being were taken into account. We chose 18 items to establish the measurement model. The items were used as indicators for the latent variables representing the students’ attitudes. The recoded items and subscales assign higher values to increasing sexual openness. As a final step, 14 scales with standardized values were constructed to distinguish the different subdimensions (see Fig 1).

#### Sexual practices

Participants reported which practices they performed with their partner during the most recent sexual intercourse, such as an active or passive form of manual sex, oral sex, vaginal sex, or anal sex. By adding one point for every practice, we constructed an additive scale with a range from 0 to 8. Additionally, the elapsed time after performing these practices was examined. This resulted in a further scale with higher values representing a more recent activity. The students could choose between *within last 4 weeks*, *within last 12 month*, *earlier*, and *never*.

#### Masturbation

We used two indicators to measure the participants’ attitude towards masturbation: the actual frequency of masturbation and their reported feelings and thoughts after masturbation. We constructed an additive scale from affirmative responses to *I was sexually satisfied*, *I enjoyed it*, *I felt relaxed*, and *I was happy*. These four items showed high correlations and were chosen as collinear representatives of positive feelings towards masturbation. The final scale ranged from 0 to 4, with the maximum value assigned to a positive view of masturbation.

#### Bisexuality

Three items were used to measure the attitude towards sexual experiences with partners of the same sex. We examined whether the students feel attracted to the same sex, could imagine a pleasurable sexual experience with partners of the same sex, and how they would describe their sexual orientation. Higher scores indicated a higher sexual flexibility, whereas lower scores implied a stronger sexual affection towards one gender.

#### Permissiveness

We constructed three different scales to measure permissiveness. First, we used the desire to have sex outside the relationship as an indicator. A second scale was based on the respondents’ definition of faithfulness and its importance in a relationship. Every disagreement to one of the following statements corresponded to one point on the scale: *My partner is unfaithful if he… flirts*/ *kisses*/ *loves*/ *has sex with someone else*/ *masturbates*/ *consumes pornography*/ *flirts on the internet*. Finally, participants were asked about the number of other people they had sex with during their relationship. If they had such experience, further points were added if they described their subsequent feelings as positive.

#### Pornography

In order to examine students’ attitudes towards pornography, we inquired whether pornography is exciting to them, when they consumed pornography on the internet last time, and with whom they did it. Additionally, we considered participants as having an increasingly open-minded attitude towards pornography the more topics they were interested in. Some examples of these topics are different combinations of actor/actress, practices, and several other contents such as sex-toys and bondage.

### Data analytic strategy

All statistical analyses were performed using STATA 12. Using confirmatory factor analysis (CFA), we investigated the existence of an underlying construct composed of the mentioned five subdimensions. CFA is able to verify existing theoretical assumptions about a latent construct with consciously attributed measurement items. The subdimensions of sexual openness were coded as latent variables and their correlations were estimated. A good CFA model fit indicates the alignment of data distribution with the latent construct tested. To check for robustness regarding different estimation algorithms maximum likelihood (without missing values: ML; with missing values: MLMV) and asymptotically distribution free (ADF) estimation were compared. ADF is a weighted least squares estimator with robust standard errors corrected for smaller samples in STATA 12 [70].

To evaluate CFA model fit different metrics are available [71–75]. χ^2^-based tests like the normal χ^2^-value or the Satorra-Bentler χ^2^-value [76] focus on the amount of deviation from the model’s assumptions in the data. This means that a high χ^2^-value and a significant test result indicate that the data deviates from the researchers’ model [77]. However, large sample sizes tend to result in rising χ^2^-values independent of model qualification [78]. χ^2^ divided by its degrees of freedom can give further information with values lower than 3 indicating a good model fit despite their statistical significance [77].

The root mean square error of approximation (RMSEA) is an alternative to χ^2^ to assess CFA model fit. It represents the discrepancy of the model assumptions and the empirical covariance matrix and yields a result between 0 and 1 with values below 0.05 indicating a good model fit.

The Tucker-Lewis-index (TLI or NNFI) analyzes the discrepancy of the χ^2^-values of the constructed model and the null model (all latent variables and measurement items in the model are connected by covariances) and reports the quality of model fit on a scale from 0 to 1 with 1 indicating the best model fit (excellent model fit > 0.95).

Similar to the RMSEA the comparative fit index (CFI) does not use the χ^2^-value to determine model fit but instead focusses on the comparison of empirical data and model produced estimations. It is corrected for sample size and results in index values from 0 to 1 with 1 indicating the best model fit (excellent model fit > 0.95).

The metrics described above were used to assess model fit in this study.

Furthermore, we tested the reliability of the latent variables with the average variance extracted (AVE) and conducted a discriminant validity test to confirm the difference of the subdimensions. A reduced model without subdimensions but instead all items as independent indicators for sexual openness was estimated to provide further information to assess the complete model. After evaluation of model fit, the subdimensions were used to calculate Cronbach’s α to get insight whether the concept of sexual openness can be appropriately described by the measured latent variables.

We cross-validated the developed concept by statistical analyses with another survey item. The item examined what kind of sexual activities the participant had already done and what she could imagine to try in future. It covered 15 different aspects, for example watching pornography, using sex toys, having sex with more than one person at the same time, using bonds/handcuffs, or using sexually stimulating substances. An additive scale with a range from 0 to 15 was constructed by adding one point for every practice which the respondent could imagine to or did engage in. We calculated this item’s correlations with the values for sexual openness based on the estimations for all subdimensions. According to our understanding of sexual openness the measure should correlate with the introduced construct of sexual openness.

## Results

### Model fit and comparison of estimation methods

We tested a five-factor model for sexual openness using confirmatory factor analysis (see Fig 1). Goodness-of-fit indices calculated for ML and MLMV resulted in similar outcomes like those for ADF. There were no considerable differences between estimation methods (see Table 1). In the following we will refer to the results of ADF estimation as it is suggested for non-normality and our sample size complies with the requirements [70, 79, 80]. All calculated fit-indices suggested an adequate model fit, CFI = 0.961, TLI = 0.947, RMSEA = 0.044, except of the χ^2^-statistic, χ^2^(68) = 174.40, *p* < .001. This is a well-known phenomenon and can be attributed to the χ^2^-test’s sensitivity to sample size and model complexity [81–83]. However, the relative χ^2^ of 2.56 (ratio of χ^2^ to its degrees of freedom) represents a good model fit. Values lower than 3 are recommended [84]. Satorra-Bentler-scaled χ^2^-statistics for ML estimation was significant providing no further evidence in favor of the model, Satorra-Bentler χ^2^(67) = 172.18, χ^2^/df = 2.57 [76]. The reduced model performed significantly worse, χ^2^(78) = 836.67, *p* < .001, CFI = 0.720, TLI = 0.673, RMSEA = 0.109, which confirms the importance of the chosen subdimensions for the construct sexual openness.

**Table 1:** Model fit for different estimation methods.

Estimation Method	χ^2^ (*df*)	χ^2^/*df*	RMSEA	95% CI	CFI	TLI
ML	184.62 (67)	2.76	0.046	[0.038, 0.054]	0.972	0.962
MLMV	195.75 (67)	2.92	0.047	[0.040, 0.055]	0.970	0.960
ADF	174.40 (68)	2.56	0.044	[0.036–0.052]	0.961	0.947

df = degrees of freedom; RMSEA = Root Mean Square Error of Approximation; CI = confidence interval; CFI = Comparative Fit Index; TLI = Tucker Lewis Index; ML = Maximum Likelihood; MLMV = Maximum Likelihood with Missing Values; ADF = Asymptotically Distribution Free.

### Factor loadings, factor reliability, and discriminant validity

All estimated factor loadings were highly significant and ranged between .60 and .92 (see Fig 1). Two outliers occurred: one item on sexual practices (sexual practices during last intercourse, factor loading = .51) and another item on pornography (pornography exciting, factor loading = .44). The highest factor loadings were identified for pornography items measuring the time period since last consumption (factor loading = .91) and the preference of different topics in pornography (factor loading = .87). Additionally, the question about the time period since performing different sexual practices showed a high factor loading (factor loading = .92).

The calculated composite reliabilities in Table 2 ranged between .69 and .85 and thus suggest an acceptable measurement (values greater .6 recommended; [85]). The smallest values were identified for the subdimensions of sexual practices and permissiveness. For sexual practices, a composite reliability of .697 was calculated. The subdimension of permissiveness consistently showed the smallest factor loadings with values between .60 and .71, resulting in the minimal composite reliability of .689.

**Table 2:** Bivariate correlations/squared correlations of all subdimensions, average variance extracted (AVE) and composite reliability.

	Sexual practices	Masturbation	Bisexuality	Permissiveness	Pornography
Sexual practices	-	.26/.07	.31/.10	.26/.07	.31/.10
Masturbation		-	.37/.14	.33/.110	.45/.20
Bisexuality			-	.47/.22	.38/.14
Permissiveness				-	.18/.03
AVE	.55	.62	.64	.43	.59
Composite reliability	.697	.762	.842	.689	.847

p < .001 for all estimated correlations.

As presented in Table 2, for correlations of the subdimensions values between .18 and .47 were calculated. The highest correlations were estimated for bisexuality and permissiveness (*r* = .47, *p* < .001), and for masturbation and pornography (*r* = .45, *p* < .001). The lowest correlation was examined for permissiveness and pornography (*r* = .18, *p* < .001). Based on the Fornell-Larcker-criterion the average variance extracted (AVE) of every factor was compared to the corresponding squared correlations with each other factor. We estimated values between .42 and .64 for the AVE. Only for the subdimension permissiveness a value lower than .5 was calculated (AVE = .42). For all other latent variables, the threshold of .5 was exceeded [86]. As the squared correlations ranged between .03 and .22. Consequently the values were lower than the respective AVE and discriminant validity was verified for all factors.

We calculated Cronbach’s α with help of the predicted values for the five subdimensions to evaluate the internal consistency of the developed construct (α = .76).

### Further construct validation

For the item, which covered 15 different sexual activities, a correlation of *r* = .58 (*p* < .001) between the amount of affirmative replies and the estimated value for openness was examined. The highest correlations with single subitems were calculated with *sex with more than one person* (*r* = .51, *p* < .001) and *using sex toys* (*r* = .42, *p* < .001), whereas the lowest values occurred for *wearing clothes of the other sex* (*r* = .02, *p* < .001) and *using sexually stimulating substances* (*r* = .13, *p* < .001).

## Discussion

The aim of this study was to examine whether attitudes towards different sexuality-related topics can be summarized in one latent construct. The results obtained by CFA suggest that the five subdimensions sexual practices, masturbation, bisexuality, permissiveness, and pornography can be subsumed to a global concept which we introduced as sexual openness. Due to the small number of indicators the value of Cronbach’s α is acceptable [87, 88] and implies high internal consistency of the five subdimensions.

ML and MLMV estimation were tested because of their widespread implementation even with non-normal distributed data [31, 61, 62]. Despite the missing normality assumption both methods resulted in an appropriate estimation. Moreover, ADF estimation was tested as recommended for non-normal distributed data. This method uses an asymptotical calculation and works best for larger sample sizes above 500 [89]. As expected, ADF estimation provided the best model fit. This indicated that all three methods are viable to estimate model fit, although the sample does not suffice with all method assumptions.

It was important to analyze the composite reliability as well as the discriminant validity to be sure that the five subdimensions represent different aspects of sexual openness. The small factor loadings for permissiveness correspond to the restrictive wording of two measurement items directly asking for wanting or having sex outside the relationship, what resulted in a small composite reliability as well. For the same reason, the threshold value for the AVE could not be obtained for permissiveness, whereas the factor loadings for all other subdimensions sufficed. Because of moral concerns most of the participants tend to negate such questions [44]. Nevertheless, emotionally faithful respondents might show a general interest in sexual contact with others and slightly increased levels of permissiveness. The small composite reliability for sexual practices alludes to its measurement with two items, one with a small factor loading of .51. This item solely examines the sexual practices for one given event, which may differ from the average. The time period since performing different practices last time allows a more comprehensive evaluation, which is reflected by its high factor loading (.92).

Guerra and Gouveia [67] constructed a measure for sexual liberalism and differentiated between two subscales: the liberalism towards the own sexuality (self scale, SS) and towards other peoples’ sexuality (other scale, OS). This is consistent with the approach of Hannon et al. [39], who estimated significant differences between the other and self scale, as participants reported more conservative attitudes towards the own behavior than towards others’. The measurement of sexual openness as outlined in the present study focused on the perception of the own sexuality and excluded the other scale because it is more a question of tolerance than of personal preferences. Sexual openness should not include general social conditions like different value systems or social peer pressure. However, external factors might have strong influence on individual levels of sexual openness.

Beyond the overall model fit the correlations of different estimated subdimensions were analyzed. High values were calculated for the correlation of pornography and masturbation representing the sexually arousing effect of sexually explicit material [57, 90–92]. The coefficient for bisexuality and permissiveness shows a more indirect relation, possibly due to the problematic measurement of permissiveness. This could be an indicator that non-heterosexual women are interested in having sexual experiences with other people of the same sex, even if they are living in a stable intimate relationship.

The strong relationship of pornography consumption and sexual behavior is an argument for the relevance this subdimension [93]. Recently, positive effects of pornography on sexuality are considered more frequently [45, 94–97]. The idea that sexually explicit media can have an educating and mind-opening effect towards different practices is supported by the results of the present study. On the other hand, the notion that a high level of pornography consumption is related to a lesser degree of sexual faith is not supported by the results of the present study. Inconsistent to findings of Wright et al. [93], at least for women, this hypothesis is questionable as the correlation between pornography and permissiveness showed low values. Additionally, the relationships between pornography and bisexuality as well as between pornography and sexual practices imply an association between consumption of sexually explicit material and the formation of the personal sexual background and self-perception. A positive connection between the frequency of purchasing pornography and the score for erotophilia was also examined by Fisher et al.[27] with the help of the SOS.

Consistent with the findings of Gerressu et al. [41], Guerra and Gouveia [67], and Guerra et al. [68] high correlations were also estimated for less obvious relations, for example masturbation and bisexuality, or masturbation and permissiveness. Altogether, the widespread moderate correlations of distinct measured variables as shown in Table 2 suggest an underlying concept beyond the five subdimensions.

The evaluation of areas of sexual exploration measured by an item, which covered attitudes towards 15 different sexual activities, provided further support for this model. Especially high correlations of sexual openness with positive answers to *sex with more than one person* and the *usage of sex toys* correspond to the theoretical arguments. However, the subitems *wearing clothes of the other sex* and *using sexually stimulating substances* showed no correlations with sexual openness. These items are related to very specific sexual preferences instead of more general aspects explained by sexual openness. Despite the wide range of the covered sexual preferences, the correlation of the estimated sexual openness and an additive scale consisting of all 15 subitems is high (*r* = .58, *p* < .001), which supports our argument. Consequently, sexual openness is able to explain the overall relation to sexuality but does not provide precise estimations of specific preferences.

### Limitations

Although this study examined important aspects and evidence for the latent construct sexual openness, it has some limitations. The data used in the present study was derived from a survey project by the Institute of Sex Research and Forensic Psychiatry at the University Medical Center Hamburg-Eppendorf [52], which contained research questions independent of the present study. Hence, the present sample had an acceptable size but the items show some deficits in wording and scale construction for the purpose of this study. This resulted in non-normal distribution in the data. The item about excitation due to pornography highlights this problem. The small factor loading refers not only to the potential difference of excitement and approving attitudes, but to the scale construction as well. Participants could choose between *yes*, *yes*, *but only some* and *no*, resulting in a share of 70% for the middle category.

Other limitations result from the sample structure: Because the UKE study is focused on the student population it was not possible to include other socioeconomic groups, which should be part of future studies on this topic.

Social desirability problems especially occurred in relation to the subdimension permissiveness. It was operationalized using three different items/scales, two of them asking for sexual contact outside the relationship. Because of an aversion to answer truthfully, the results could be biased and a more sensitive wording would have been appropriate. Further conceptual work is necessary to determine whether unfaithful behavior should be part of sexual openness at all. Reflecting these issues the calculated statistical parameters were worst for permissiveness.

## Conclusion and future prospects

The present study aimed to demonstrate the existence of sexual openness as the underlying concept of apparently distinct attitudes towards sexual topics. The conceptual work was influenced by existing constructs like erotophilia and sexual liberalism and focused on the five subdimensions sexual practices, masturbation, bisexuality, permissiveness, and pornography. The consideration of a five-factor model led to a balanced emphasis on the different subdimensions in comparison to previous constructs.

The current study provides an impulse for further research to unify former concepts of attitudes towards sexuality. Further investigation is necessary, and the idea of sexual openness should be validated by items deliberately constructed to measure its subdimensions. The usage of more sensitive scales could provide more detailed information about the relationships of the five subdimensions. For future studies it would be valuable to cross-validate these findings with a new sample, which includes women of different age groups and education levels. Additionally, the examination of the concept of sexual openness in men or single people is of high interest.

Sexual openness is a useful variable for developing theories to explain sexual behavior, problems, and attitudes. Its subdimensions are influenced by the conception of moral and religion, sexual subjectivity, body image, communication, and a broad range of experiences [15, 28, 82, 83, 98–100]. Conversely, they interact with variables such as sexual communication or satisfaction [29, 31, 101]. Therefore, sexual openness is expected to have a high influence on individual well-being and happiness. Further research on this topic serves to reach a more comprehensive understanding of human sexual functioning and can provide insights for clinical application.
